# Memoranda Respecting Some Investigations into the Manner in Which the Nutrition of the Fœtus Is Effected

**Published:** 1827-09

**Authors:** C. Lee


*Memoranda respecting some Investigations into the Manner in which the Nutrition of the Fœtus is effected, which have recently been made by* R. LEE, M.D.

DR. LEE, from some inquiries in which he has lately been engaged, has been led to conclude that a process of digestion goes on in the upper intestines of the fœtus, similar in kind to that which takes place after birth, and consequently that the nutrition and growth of the fœtus are chiefly, or perhaps entirely, effected in this manner. He has ascertained that the stomach of the fœtus, from three to nine months old, invariably contains a transparent mucous and acid fluid, but never the smallest admixture of albuminous or nutritious matter; while, on the other hand, the upper half of the small intestines always contains a yellowish or orange-coloured pultaceous mass, which, in appearance as well as chemical composition, resembles exactly the chyme of the adult: in a word, that it is nearly pure albumen. The contents of the lower half of the small intestines contain a much smaller proportion of albumen than those of the upper half, and the matters gradually assume more and more the characters of the contents of the large bowels, in proportion as the distance from the valve of the colon diminishes. The meconium is confined exclusively to the great intestine, and is generally possessed of alkaline properties, while the contents of the small intestines above alluded to are almost invariably acid. The meconium appears further to be merely excrementitious, as no albuminous or other nutritive principle can be detected in it.

It is remarkable that a fluid resembling that contained in the duodenum in appearance and in chemical composition,—in other words, pure albumen,—has likewise been detected in the hepatic duct of the fœtus; from which circumstance, and from the orange-coloured fluid first appearing in the duodenum, near the opening of the common duct, it may be inferred that the liver of the fœtus secretes the nutritious matter, which in no instance has been found wanting in the upper part of the small intestines. It may likewise fairly be concluded that the nutrition of the fœtus is accomplished partly, if not entirely, through the absorption of the albuminous matter so secreted from the intestinal canal, as in the adult Lacteals, however, have not yet been discovered in the human fœtus, although they have in a fœtal calf of six months.

The small intestines in the fœtus are generally much more red and vascular than the great intestines; but this is particularly striking with regard to the mucous membrane of the duodenum and jejunum, to which the albuminous matter closely adheres, and which affords a strong presumptive proof that they perform some more active and important function. The great size of the liver in the fœtus has long been matter of observation; while the function which it performed has been a source of frequent and unsatisfactory conjecture. It is a remarkable fact, too, that while almost every other organ has been wanting in the various kinds of human monstrosity, no instance has been recorded in which the liver has been absent. Its great size, its large supply of blood, and its invariable presence, accord with the hypothesis above described, which attributes to it the office of separating albumen from the blood during fœtal existence. This idea, especially the fact first mentioned of the stomach containing no nutritious matter, militates against the opinion of some of the latest and ablest physiologists, who have maintained that the nutrition of the fœtus, as of the adult, is effected by the matters received into this viscus.

In conducting the chemical part of these interesting investigations, Dr. Lee has had the able assistance of Dr. Prout.

*London; July* 1827.

---

