# Erratum

**Published:** 1827-10

**Authors:** 


Erratum.—In the paper on the Nutrition of the Fœtus, in our last Number, for "Dr. C. Lee," read "Dr. Robert Lee."

